# Optimal Coil Packing Density for Aneurysmal Complete Obliteration in Flow Diversion Stenting: A Retrospective Study

**DOI:** 10.1161/SVIN.125.001751

**Published:** 2025-07-03

**Authors:** Ryosuke Maeoka, Hiroyuki Ohnishi, Shohei Yokoyama, Masashi Kotsugi, Ryosuke Matsuda, Shuichi Yamada, Ichiro Nakagawa

**Affiliations:** ^1^ Department of Neurosurgery Nara Medical University Nara Japan; ^2^ Department of Neurosurgery Ohnishi Neurological Center Hyogo Japan

**Keywords:** coil embolization, flow diverter stent, large or giant aneurysms, packing density, volume embolization ratio

## Abstract

**Background:**

Flow diverter stent (FDS) deployment with coil embolization (CE) achieves high aneurysmal occlusion rates. However, no established guidelines exist for the optimal packing density (PD) when treating large or giant aneurysms. This study aimed to investigate the optimal PD in FDS deployment with CE.

**Methods:**

This retrospective multicenter study involved 97 consecutive patients with unruptured large or giant (≥ 10 mm) aneurysms treated with FDS deployment alone or with FDS and CE, between August 2019 and December 2023. We calculated the volume embolization ratio as the coil PD using Angiosuite Neuro Edition ver. 10.03. We defined O'Kelly–Marotta grading scale D as complete obliteration (CO) at 6 months after deployment and analyzed the associations between the PD and aneurysmal CO.

**Results:**

Seventeen patients (means ± SD age, 53.8±9.94 years; 14 [82.4%] female patients) in the FDS with CE group, and 18 patients (63.6±12.4 years; 14 [77.8%] female patients) in the FDS alone group were included for analysis. CO was achieved in 14 (82.3%) patients in the FDS with CE group and 9 (50.0%) in the FDS alone group (*P* = 0.08). volume embolization ratio (≥ 8.9%) was significantly associated with CO in the univariable analysis (*P* = 0.01; odds ratio: 14.3 [95% CI: 1.57–129.9]) and the multivariable analysis (*P* = 0.02; odds ratio, 10.5 [95% CI: 1.09–100.7]).

**Conclusion:**

It may be unnecessary to continue CE after the PD has reached 8.9% volume embolization ratio when treating large or giant intracranial aneurysms with combined FDS and CE.

## Graphical abstract

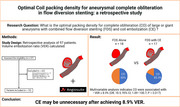

**Nonstandard Abbreviations and Acronyms**

CE: coil embolization
CO: complete obliteration
DSA: digital subtraction angiography
EVT: endovascular treatment
FDS: flow diverter stents
OKM: O'Kelly–Marotta
PD: packing density
PED: pipeline embolization device
VER: volume embolization ratio

**Clinical Perspective**

- This is the first study to calculate the volume embolization ratio as coil packing density affordably and flexibly using freely‐available software for everyone to predict complete obliteration of large or giant aneurysms treated with flow diverter stent deployment with coil embolization.
- Because large or giant aneurysms can cause mass effect and cranial nerve palsies, worsened by coil mass, it is important for all patients and all clinicians to pursue an optimal packing density during endovascular treatment for both cost‐effectiveness and complication prevention perspectives.

Flow diverter stents (FDS) are a pioneering advancement in the management of intracranial aneurysms. FDS deployment achieves higher occlusion rates and lower retreatment rates for giant aneurysms than coil embolization (CE).^1^, ^2^ FDS deployment represents a contemporary and notable treatment for the management of large or giant cerebral aneurysms.^3^ FDS are densely porous metallic stents that are deployed within the parent artery, enveloping the neck of the aneurysm, with the aim of achieving hemodynamic alterations. Regarding pipeline embolization device (PED) deployment, the combined deployment of FDS and CE provides greater efficacy in achieving aneurysmal occlusion than PED deployment only.^4^, ^5^ Some interventionalists combine FDS deployment and CE to achieve higher aneurysmal occlusion rates or to prevent delayed aneurysmal rupture. However, for FDS deployment with CE, there is currently no established guideline regarding the optimal packing density (PD) for large or giant aneurysms, so loose‐packing CE is common. To the best of our knowledge, few studies have investigated the correlation between coil PD and the aneurysmal occlusion rate in cerebral aneurysms treated with combined FDS deployment and CE.^6^ Using computational fluid dynamics analysis and a finite element method based workflow, Zhang et al reported that in cases of FDS with CE, PD reached an average of 7.06%; therefore, it may be unnecessary to continue CE thereafter.^6^ In the study, the reduction in the velocity ratio, and metal coverage, with PEDs were calculated using computational fluid dynamics analysis, with a specialized fee‐based software. Damiano et al also simulated the optimal PD of coils after FDS deployment using a finite element method‐based workflow and proved that there is no hemodynamic impact of additional coils until the PD exceeds 11%.^7^ We consider that these methods are complicated, expensive, and not available to all. Therefore, to predict complete obliteration (CO) of aneurysms treated with FDS, in this study, we analyzed the factors associated with CO, including the PD, which was calculated affordably and flexibly using freely available software.

## Methods

The data that support the findings of this study are available from the corresponding author upon reasonable request. The corresponding author had full access to all the data in the study and takes responsibility for its integrity and the data analysis.

### Ethics Approval

This retrospective multicenter study was approved by the institutional ethics committees of Nara Medical University (Approval number: 3892) and Ohnishi Neurological Center. Informed consent was obtained as an opt‐out on both institutional websites.

### Study Design

This retrospective multicenter observational study adhered to the Strengthening the Reporting of Observational Studies in Epidemiology criteria. This study assessed the rate of CO at 6 months from endovascular treatment (EVT) and the volume embolization ratio (VER) as coil PD in patients treated with FDS deployment for large or giant cerebral aneurysms from 2019 to 2023. Aneurysm occlusion was classified based on the O'Kelly–Marotta (OKM) grading scale.^8^ Postoperative digital subtraction angiography (DSA) was performed at 6 months after EVT to assess the OKM grading scale. We defined the OKM grading scale D as CO, at 6 months after EVT. DSA was performed using a biplane angiographic system (Axiom Artis Q or icono; Siemens Healthineers, Erlangen, Germany). The size of aneurysms was adjudicated by local site investigators at each hospital. We used the freely available software, Angiosuite Neuro Edition, ver. 10.03 (Cascade Medical LLC, Knoxville, TN, USA) to calculate the VER from 3‐dimensional rotational angiography. In the freely available software, aneurysm volume was calculated as π×(H×W×D)/6, where H, W, and D representing the largest diameters of the aneurysm in the height, width, and depth, respectively. Subsequently, all coils deployed into the aneurysm were selected, after which the VER was automatically calculated.

### Study Population and Clinical Data

In this retrospective study, data were reviewed for 97 consecutive patients with unruptured cerebral aneurysms treated with FDS deployment, either alone or with CE, between August 2019 and December 2023 at Nara Medical University and Ohnishi Neurological Center. Large or giant aneurysms (maximum diameter ≥ 10 mm), and aneurysms treated with a single FDS were included. The exclusion criteria were as follows: (1) recurrent aneurysms, (2) no follow‐up DSA images, (3) treatment with telescopic stenting, and (4) treated aneurysms that measured <10 mm. The electronic medical records of each patient were reviewed, and data were obtained for age at EVT, sex, medications, date of EVT, risk factors for cardiovascular disease, the OKM grading scale as the radiographical outcome, the number of coils, vascular anatomical characteristics, and VER as coil PD.

### Study Outcomes

The primary endpoint was the CO rate. The secondary endpoints were perioperative ischemic stroke and cerebral hyperperfusion syndrome following treatment with FDS and delayed aneurysmal rupture.

### Antiplatelet Therapy

All patients received dual antiplatelet therapy with aspirin (100 mg per day) and clopidogrel (75 mg per day) for at least 10 days prior to EVT. Platelet inhibition levels were analyzed using the VerifyNow aspirin assay (in aspirin reaction units) and P2Y12 assay (in P2Y12 reaction units) (Accumetrics, San Diego, CA, USA) on the 2 days before EVT. The target reaction units of aspirin and clopidogrel were < 550 and < 200, respectively. When the reaction units did not reach the target values, clopidogrel was changed to prasugrel (loading dose: 20 mg; maintenance dose: 3.75 mg daily). We then analyzed platelet inhibition levels the day before EVT. The target values were reached in all cases. Postoperative antiplatelet therapy was continued as aspirin (100 mg per day) and the maintenance dose of clopidogrel (75 mg per day) or prasugrel (3.75 mg per day) for 6 months after EVT. After 6 months, clopidogrel or prasugrel therapy was discontinued, and aspirin alone was used, on the basis of the angiography results.

### Endovascular Treatment

All patients were treated under general anesthesia. Patients underwent FDS deployment alone or with CE during a single procedure. Following arterial access, to achieve an activated clotting time of > 250 seconds, patients received intravenous heparin. In patients who underwent FDS deployment alone, procedures were performed via right femoral artery access. Access was obtained using a 5‐Fr or 6‐Fr guiding sheath, which was placed in the cervical segment of the artery leading to the target intracranial arteries. Then, a 5‐Fr distal access catheter was placed in the distal target intracranial artery. Subsequently, a 0.027‐inch microcatheter was navigated over a 0.012 to 0.014‐inch outer diameter microwire to position the microcatheter across the target aneurysmal neck. Finally, FDS deployment was performed using a combination of unsheathing the device and pushing the stent out of the microcatheter. Patients who underwent FDS deployment with CE were treated using the microcatheter jailing technique. In these patients, procedures were performed via bilateral femoral artery access. FDS deployment was as described via right femoral artery access. Additionally, a 5‐Fr guiding catheter was placed in the target intracranial artery via left femoral artery. Then, coils were subsequently deployed through the positioned microcatheter in an aneurysm. Specifically, a 0.027‐inch microcatheter was positioned distal to the aneurysmal neck via right femoral artery at first, then a second microcatheter paralleling to the distal access catheter was positioned into the aneurysmal sac via left femoral artery. Next, the FDS was deployed from the distal landing point, effectively covering the neck of the aneurysm. Subsequently, coils were deployed through the previously positioned second microcatheter within the aneurysm. Following CE, the coiling microcatheter was carefully withdrawn from the aneurysm without disrupting the structure. Clinically, the choice of FDS deployment alone or with CE was at the treating physician's discretion, considering the anatomical evaluation of the aneurysm(s), the patient's comorbidities, and the risk of recurrence. In patients who underwent CE, the number of coils was also at the treating physician's discretion.

### Statistical Analysis

Univariable analysis of the investigated parameters was performed to determine which were significantly different between the occluded and recurrent groups. Categorical variables were reported as number (percentage), and continuous variables were reported as median with interquartile range or mean ±SD. Statistical analysis was performed with the chi‐square test for categorical variables and with the Wilcoxon rank sum test for continuous variables. Receiver operating characteristic (ROC) curves were constructed to determine the optimal cutoff values for age, maximum aneurysmal diameter, size of the aneurysmal neck, number of coils, and VER to predict the OKM grading scale, as the radiographical outcome. ROC curve analysis involves drawing a plot of sensitivity (true positive rate) by 1−specificity (false positive rate) for continuous variables by dichotomizing patients as with or without CO. We used a ROC curve analysis to accurately identify the cut‐off points. Additionally, parameters that were statistically significant in the univariable analysis and considered risk factors on the basis of previous research were included in the multivariable logistic regression analysis to remove co‐dependence and identify independently significant parameters associated with CO.^5^, ^9^ The regression involved a backward conditional stepwise method with a removal criterion of *P*>0.1. A 2‐sided *P* value of <0.05 was considered statistically significant. Because the number of aneurysmal complete obliteration as the primary endpoint was 23 cases in this study, 2 explanatory variables were used. VER is not determined by the number of coils, rather is calculated by the diameter and length of the coils.^10^ We considered the presence and number of coils to be similar, selecting VER as an explanatory variable for the multivariable logistic regression analysis. We compared the frequencies of the risk factors between the FDS with CE group and the FDS alone group. We then constructed logistic regression models to estimate odds ratios (ORs) with 95% CIs for the primary and secondary endpoints. A post hoc analysis was performed to detect statistical power using the VER cutoff points calculated from the ROC curves. Descriptive and frequency analyses were performed, and comparisons were made using JMP Pro 17.0.0 software (SAS Institute, Cary, NC, USA).

## Results

### Baseline Characteristics of the Patients

Ninety‐seven patients with cerebral large or giant unruptured aneurysms underwent surgery during the study period. Sixty‐two patients were excluded in accordance with the exclusion criteria, as follows: 7 patients with recurrent aneurysms, 4 patients with no follow‐up DSA images, 6 patients treated with telescopic stenting, and 45 patients with aneurysms that measured < 10 mm. After applying the exclusion criteria, 35 patients were included in this study (Figure). The baseline characteristics of those 35 patients are shown in Table 1. Seventeen patients (mean ± SD age, 53.8±9.94 years; 14 [82.4%] female patients) in the FDS with CE group and 18 patients (mean ± SD age, 63.6±12.4 years; 14 [77.8%] female patients) in the FDS alone group were included for analysis (*P* = 0.01). The proportion of female patients was not significantly different between the 2 groups (*P* = 1.00). Dyslipidemia was significantly less frequent in the FDS with CE group (*P* = 0.01). Additionally, no differences in antiplatelets medication regimen were noted between the FDS with CE group and the FDS alone group (*P* = 0.40).[Fig svi213038-fig-0001]

**Figure 1 svi213038-fig-0001:**
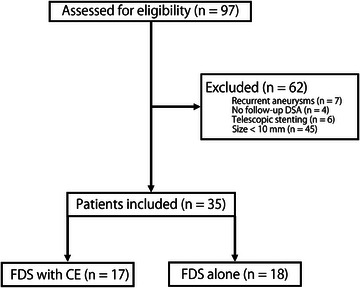
**Flow diagram of the current retrospective study**. CE indicates coil embolization; DSA, digital subtraction angiography; and FDS, flow diverter stent.

**Table 1:** Baseline Characteristics of the Patients

Category	Number			*P* value
	Total cases (n = 35)	FDS alone (n = 18)	FDS with CE (n = 17)	
Demographic characteristic				
Age, y (mean±SD)	58.8 ± 12.1	63.6 ± 12.4	53.8 ± 9.94	0.01
Female patients, n (%)	28 (80.0)	14 (77.8)	14 (82.4)	1.00
Hypertension, n (%)	24 (68.6)	14 (77.8)	10 (58.8)	0.29
Dyslipidemia, n (%)	7 (17.1)	7 (38.9)	0 (0)	0.01
Diabetes, n (%)	3 (8.6)	1 (5.6)	2 (11.8)	0.60
Current smoker, n (%)	10 (28.6)	5 (27.8)	5 (29.4)	1.00
Chronic kidney disease, n (%)	6 (17.1)	4 (22.2)	2 (11.8)	0.66
Antiplatelet medication regimen				0.40
	Aspirin + clopidogrel, n (%)	28 (80.0)	13 (72.2)	15 (88.2)
	Aspirin + prasugrel, n (%)	7 (20.0)	5 (27.8)	2 (11.8)

FDS indicates flow diverter stent.

### Aneurysm Characteristics

The differences in aneurysm characteristics between the FDS with CE group and the FDS alone group are shown in Table 2. No differences in aneurysmal size were observed between the FDS with CE group (14.3±4.28 mm) and the FDS alone group (15.5±5.26 mm) (*P* = 0.45). There was a tendency toward aneurysmal neck size in the FDS alone group (8.58±2.09 mm) compared with the FDS with CE group (7.03±2.77 mm) (*P* = 0.06). Aneurysms located intradurally (*P*<0.0001) and on the outer curvature (*P* = 0.02) were significantly more likely to be treated with combined FDS and CE.

**Table 2:** Aneurysm Characteristics

Category	Number			*P* value
	Total cases (n = 35)	FDS alone (n = 18)	FDS with CE (n = 17)	
Aneurysmal characteristics (median (mean±SD))				
Aneurysmal size, mm	12.9 (14.9 ± 4.78)	13.3 (15.5 ± 5.26)	12.0 (14.3 ± 4.28)	0.45
Neck size (mm)	8.05 (7.82 ± 2.53)	8.62 (8.58 ± 2.09)	5.69 (7.03 ± 2.77)	0.72
Intradural, n (%)	21 (60.0)	4 (22.2)	17 (100)	<0.0001
Outer curvature, n (%)	26 (74.3)	10 (55.6)	16 (94.1)	0.02
Involving an artery branch, n (%)	7 (20.0)	2 (11.1)	5 (29.4)	0.23

FDS, indicates flow diverter stent.

### Treatment and Procedure Outcomes

The differences about aneurysm characteristics between the FDS with CE group and the FDS alone group are shown in Table 3. FDS deployment was technically successful in 100% of the cases. Of the patients with DSA follow‐up images at 6 months after EVT, 23 patients (65.7%) were classified as CO (the OKM grading scale D), occlusion was deemed incomplete in 3 patients (8.6%) (1 each of the OKM grading scale B1, 2, and 3, respectively), and 9 patients (25.7%) had a neck remnant (1 for the OKM grading scale C1, 4 for C2, and 4 for C3, respectively). CO was achieved (the OKM grading scale D) in 14 (82.3%) patients in the FDS with CE group and 9 (50.0%) in the FDS alone group; thus, there was tendency toward a higher prevalence of CO in the FDS with CE group (*P* = 0.08). There were no significant differences between the groups for procedural ischemic stroke (*P* = 1.00). No patients in either group developed cerebral hyperperfusion syndrome, delayed aneurysmal rupture, or mass effect symptoms following treatment with FDS. The ROC curve analyses for age and VER showed areas under the curve of 0.737 and 0.745, respectively, and the best cutoff values were 56 years and 8.9%, with a sensitivity of 60.9% and 56.5%, and specificity of 83.3% and 91.7%, respectively (Table 4).

**Table 3:** Procedure Characteristics

Category	Number			*P* value
	Total cases (n = 35)	FDS alone (n = 18)	FDS with CE (n = 17)	
Successful FDS deployment, n (%)	35 (100)	18 (100)	17 (100)	‐
Complete wall apposition, n (%)	35 (100)	18 (100)	17 (100)	‐
FDS used, n (%)				1.00
PED	31 (88.6)	16 (88.9)	15 (88.2)	
Others (FRED, Surpass)	4 (11.4)	2 (11.1)	2 (11.8)	
The OKM grading scale, n (%)				
B				
1	1 (2.9)	0 (0)	1 (5.9)	
2	1 (2.9)	0 (0)	1 (5.9)	
3	1 (2.9)	1 (5.6)	0 (0)	
C				
1	1 (2.9)	1 (5.6)	0 (0)	
2	4 (11.4)	3 (16.7)	1 (5.9)	
3	4 (11.4)	4 (22.2)	0 (0)	
D				
	23 (65.7)	9 (50.0)	14 (82.3)	0.08
Perioperative ischemic stroke, n (%)	1 (2.9)	1 (5.6)	0 (0)	1.00
Hyperperfusion syndrome, n (%)	0 (0)	0 (0)	0 (0)	‐
Delayed aneurysmal rupture, n (%)	0 (0)	0 (0)	0 (0)	‐
Mass effect symptoms, n (%)	0 (0)	0 (0)	0 (0)	‐

CE indicates coil embolization; FDS, flow diverter stent; FRED, flow‐redirection endoluminal device; OKM, O'Kelly–Marotta; and PED, pipeline embolization device.

**Table 4:** ROC Curves to Predict Aneurysmal Complete Obliteration (n = 35)

Category	Cutoff point	Sensitivity	Specificity	Accuracy	AUC
Age, y	56	0.609	0.833	0.343	0.737
Maximum diameter, mm	15.82	0.435	0.750	0.543	0.558
Neck size, mm	8.65	0.6956	0.500	0.371	0.533
Number of coils, n	2	0.609	0.833	0.686	0.728
VER, %	8.9	0.565	0.917	0.686	0.745

AUC indicates area under the ROC curve; ROC, receiver operating characteristic; and VER, volume embolization ratio.

### Predictors of Achieving Aneurysmal CO

The predictors of achieving aneurysmal CO are shown in Table 5. In the univariable analysis, the significant factors associated with CO were VER (≥ 8.9%) (OR, 14.3 [95% CI, 1.57–129.9]; *P* = 0.01), the use of 2 or more coils (OR, 7.78 [95% CI, 1.37–44.0]; *P* = 0.03), and advanced age (≥ 56 years) (OR, 0.15 [95% CI, 0.03–0.87]; *P* = 0.03). CE tended to be associated with aneurysmal CO at 6 months after EVT (OR, 4.67 [95% CI, 0.99–22.0]; *P* = 0.08). In the multivariable analysis, VER (≥ 8.9%) was significantly associated with aneurysmal CO at 6 months after EVT (OR, 10.5 [95% CI, 1.09–100.7]; *P* = 0.017).

**Table 5:** Predictors of Achieving Aneurysmal Complete Obliteration at 6 Months After EVT

Predictive variable	Univariable analysis			Multivariable analysis		
Demographic characteristic	ORs	95% CIs	*p* value	ORs	95% CIs	*P* value
Age ≥ 56 y	0.15	0.03–0.87	0.03	0.24	0.04–1.53	0.11
Sex (female)	1.39	0.23–8.51	1.00			
Hypertension	0.63	0.13–2.98	0.71			
Dyslipidemia	0.30	0.05–1.65	0.20			
Diabetes	1.05	0.09–12.9	1.00			
Current smoker	1.31	0.27–6.37	1.00			
Chronic kidney disease	0.45	0.08–2.68	0.39			
Aneurysmal characteristics						
Aneurysmal size ≥ 15.82 mm	2.31	0.49–10.8	0.46			
Neck size ≥ 8.65 mm	0.44	0.10–1.84	0.30			
Outer curvature	1.80	0.38–8.53	0.69			
Involving an artery branch	1.39	0.23–8.51	1.00			
Coil embolization	4.67	0.99–22.0	0.08			
Number of coils ≥2	7.78	1.37–44.0	0.03			
Volume embolization ratio ≥ 8.9%	14.3	1.57–129.9	0.01	10.5	1.09–100.7	0.017

EVT indicates endovascular treatment; and OR, odds ratio.

## Discussion

In this study, we detected the predictive factors associated with aneurysmal CO. To our knowledge, this is the first study to detect the optimal PD using VER calculated with freely‐available software. Coil packing beyond a VER of 8.9% might be unnecessary, as CO of large or giant aneurysms is more easily achieved at a PD of VER = 8.9% in cases treated with FDS deployment with CE.

### Optimal PD

Little is known about the minimum PD required to resolve unruptured intracranial large or giant aneurysms.^6^, ^7^ Additionally, the optimal PD in previous studies was calculated with a specialized fee‐based software.^6^, ^7^ Therefore, the goal of the current study was to investigate a simple method to determine the optimal PD of aneurysmal occlusion with coils using freely available software when FDS deployment is combined with CE. Some studies have revealed the optimal PD of coils after FDS deployment. Zhang et al reported that the optimal PD was 7.06%,^6^ and Damiano et al indicated that the optimal PD was 11%.^7^ However, these studies involved idealistic models or clinical models simulated using a specialized fee‐based software. In the present study, in which freely available software was used, we determined the optimal PD as VER = 8.9%, which is comparable to the values in previous studies. Our findings of the optimal PD are also comparable to values in previous studies. In the present study, we used Angiosuite Neuro Edition ver.10.03 to calculate the VER, which resulted in the following values: 56.5% sensitivity, 91.7% specificity, and 68.6% accuracy. Moreover, the statistical power of VER = 8.9% was 70.1%. Notably, this software is accessible to all users and allows ongoing calculation of VER; thus, avoiding unnecessary CE. In the present study, we reviewed data for cerebral large or giant (≥ 10 mm) unruptured aneurysms. Aneurysms with a width of 10 mm pose greater challenges in treatment and are more predisposed to recurrence after EVT compared with aneurysms with a width of < 10 mm.^11^, ^12^ Compared with CE, EVT using FDS achieves high rates of occlusion when addressing intricate, massive aneurysms, while also reducing the necessity for retreatment.^1^, ^2^ Additionally, Park et al reported a significantly reduced retreatment rate in the cohort that received combined PED deployment and CE, in contrast to those treated with PEDs alone.^13^ Some retrospective studies revealed that the occlusion rate associated with PED deployment with CE was notably higher compared with PEDs alone.^14^, ^15^ Moreover, Wali et al reported that the cost of endovascular retreatment after the initial EVT matched that of CE in a model of large or giant unruptured aneurysms.^16^ Achieving aneurysmal CO with only the initial EVT is cost effective and advantageous. Moreover, reducing the number of coils in a single EVT is understandably beneficial from a medical economics perspective.

Notably, patients with large or giant aneurysms often present with a mass effect and cranial nerve palsy affecting the optic nerve and ocular cranial nerves, especially in cavernous sinus lesions. The mass effect caused by the coil mass may not only worsen the underlying mass effect but also be a factor in worsening cranial nerve palsy.^17^ Additionally, Akiyama et al reported a low rate of symptom improvement when the VER was high, particularly at VER ≥ 13%.^18^ Moreover, Wang et al found that in cases of symptomatic aneurysms treated with FDS deployment combined with CE and a loose PD (VER < 12%), there were significant rates of aneurysmal regression and symptom improvement.^19^ Consequently, especially in cases of large or giant aneurysms in patients with cranial nerve palsy, it might be effective to aim for a PD with VER 7.06%–12%. Therefore, our finding of an optimal PD of VER = 8.9% is reasonable. We considered that pursuing an optimal PD may be useful from both cost‐effectiveness and complication prevention perspectives, prompting this study. In this study, we observed that aneurysmal CO could not be achieved with 91.7% specificity at VER < 8.9% when large or giant aneurysms were treated with FDS deployment with CE. Additionally, CO was 10.5 times more likely when VER exceeded this threshold compared with FDS alone.

### Safety of FDS with CE

Complete aneurysmal occlusion has been achieved after PED deployment combined with CE, without notable occurrences of hemorrhagic or thromboembolic complications.^20^, ^21^ Other reports have also indicated no significant differences between the 2 groups (PED alone group and PED with adjunctive CE group) for procedural ischemic stroke and hemorrhage and delayed aneurysmal rupture.^13^, ^15^ FDS with CE is considered safe. No significant differences were observed in the present study for procedural ischemic stroke, nor were there instances of cerebral hyperperfusion syndrome or delayed aneurysmal rupture.

### Limitations

Our study has limitations. First, the follow‐up period was short. Currently, long‐term clinical and angiographic follow‐up analysis extending beyond 1 year is unavailable and remains a subject for future investigation. Second, this was a retrospective study with local site adjudication, and selection bias was present regarding the choice of combined FDS with CE or FDS alone. This is a particular concern regarding the determination of the optimal quantity of the parent artery to occlude with coils. Additionally, due to the study design, many cases were excluded; randomized controlled trials with core lab adjudication are required. Finally, the major shortcoming of the current study is that it involved only 2 centers and included a small number of cases, which made objective assessment difficult. Therefore, a larger number of cases must be accumulated and evaluated further.

## Conclusion

We believe that the results of the present study may help clinicians identify the appropriate PD in aneurysm coiling. Additionally, it may be unnecessary to continue packing after the PD has reached 8.9% VER when using combined FDS and CE.

## Conflict of Interest Statement

None.

## Sources of Funding

This research did not receive any specific grant from funding agencies in the public, private, commercial, or not‐for‐profit sectors.
